# Oral Prophylaxis

**Published:** 1901-12

**Authors:** D. D. Smith

**Affiliations:** Philadelphia


### International Dental Journal.

Vol. XXII. December, 1901. No. 12

### Original Communications.[1]

### ORAL PROPHYLAXIS.[2]

BY D. D. SMITH, D.D.S., M.D., PHILADELPHIA.

In the summer of 1882 the Pennsylvania State Dental Society came over into this great Empire State of New York and held its annual meeting in Chautauqua. I never understood the true reason of this unusual procedure, the convening of a State society outside the boundaries of its own State, but I remember the proceedings as well as the discussions at those meetings, and plainly recall some of the participants.

It was my part and privilege to advocate for the first time the practice of utilizing roots and sound teeth, even to the destruction and extirpation of normal pulps, where necessary to make them available as supports for sustaining artificial teeth or bridge-work.

I had at the time little comprehension of crown- and bridge-work as exhibited at its best to-day, but I felt and knew that the destruction of the pulp did not mean the destruction of the *tooth,* and whilst the practice was without the test of great experience at that time, it yet had the underlying support of reason as well as

[1] The editor and publishers are not responsible for the views of authors of papers published in this department, nor for any claim to novelty, or otherwise, that may be made by them. No papers will be received for this department that have appeared in any other journal published in the country.

[2] Read before the New York Institute of Stomatology, June 4, 1901.

physiological principles. I shall probably never forget the discredited, crestfallen state in which I emerged from these discussions for my somewhat timid advocacy of what was then a most unpopular and heterodox practice. Nineteen years have rolled around, and what a change in sentiment! The Central Dental Association of New Jersey, at its meeting in January, 1901, awakened to a kind of symposium discussion of practically the same subject: "Is the dental pulp necessary in the tooth of the adult?"

The consensus of opinion, as I read the articles in the March number of *Items of Interest,* leans decidedly in favor of the theories and methods which were so severely denounced by men of prominence at the meeting of the Pennsylvania State Dental Society in 1882.

This first attempt at a comprehensive discussion of the value of the pulp in adult life, as given in the articles alluded to, fails in each individual instance to grasp the vital issue. The point of real vital importance is not touched, and as a consequence this discussion fails in the unification of any fixed or definite conclusions or teachings.

To arrive at any adequate conception of the value of the pulp in adult life, there must first be a better and clearer understanding of what constitutes *tooth-life.* And in order to do this, the ordinary complex division of the tooth should be abandoned, and the tooth resolved into its natural and distinct parts, crown and root, with full recognition of its two separate and distinct sources of life and nutrition. There must also be recognition of the fact that the *root* of the tooth, and not its crown, is the real, vital, living anatomy. A genuine living, functional tooth can be maintained without any portion of its natural crown; but a crown, no matter how perfect it may be, is entirely valueless unless supported on a living root. It must be recognized that the pulp-life which goes alone to the crown in its entirety—to its dentine and enamel and to the dentine of the root—is entirely separate and distinct from the life of the cemental tissue of the root; and that this cemental life is supplied wholly by the pericementum and its adjuvants. The crowns of teeth may be slightly affected or largely destroyed by decay, or even entirely obliterated, without appreciable interference with the integrity, utility, or even the æsthetics of the real tooth.

The fact that a tooth with a living pulp and an unimpeachable crown may, and often does, become entirely valueless through disease in cemental and pericemental tissue, fails of recognition even in the discussions of this New Jersey Society.

What shall become of a pulp, either through disease or interference, is a matter of trifling import so long as the cemental and pericemental life remains intact. *But destroy the pericemental life, and you destroy the tooth.*

Again, to form a just estimate of the value of the pulp in adult life, there must be an appreciation of the influence of misdirected pulp-activity in converting the cementum of the root into denser structure, as frequently results in adult life. Cementum, that all-important tissue of the root, is thickest and most abundant and most vascular in young life; and because of this the integrity of the tooth is best maintained at that period. Teeth with *dense* cemental tissue, sparsely distributed over the roots, are found in adult life only; and this denser cemental tissue is always, we believe, on teeth with living pulps; whilst abundant and properly distributed cemental tissue, so necessary to the conservation of the tooth, is found on young teeth generally, and on teeth and roots with devitalized pulps.

I have before made the assertion, and it stands without contradiction, that pyorrhœa rarely or never begins in *young* adults; neither is it found in connection with devitalized teeth where the devitalization occurred in young life. These conditions existing, it necessarily follows that whilst through destruction of the pulp there may be inconsequent injury or damage to the crown, there is yet no interference with cemental and pericemental life and consequently no injury to the real, living, and most important part of the tooth. These phenomena and deductions, as addenda to the discussions on "the value of the pulp in adult life," are not speculative merely, but they bear important relations to one phase in particular of oral prophylaxis.

For years it has been, and still is, a settled conviction with me that dentistry, with all its conceded progress and improvements, has yet neither done its most important work nor met the reasonable demand of suffering humanity. It has occupied itself in mechanics and mechanism; in the change and improvement in instruments and appliances; in the presentation of materials and methods, and has left the more important matters of cause and prevention almost wholly without investigation.

Time and again we have changed our advocacy of this method and that material; running this procedure through the whole gamut of amalgams and cements; of gold, crystal, soft and cohesive; using it in mats, rolls, and sheets. We have filled with mallets innumerable, contouring without matrix and with. We have followed professional instruction, and made "cavity preparation" the most important part of our operations. We have compounded fillings and protected cervical margins with amalgams, tin- and soft foil, and yet, as if in very mockery of our mechanical efforts, decay and redecay have marched steadily forward; and to-day the most popular theme for dental discussion is not prevention by removal of cause, but "prevention by extension." In an article of over thirteen pages in *Items of Interest* for May, 1901, E. K. Wedelstaedt, D.D.S., gives to the profession and to the world the methods which, he says, "I teach and follow, and which I feel should be followed by all who have the welfare of those whom we serve at heart." Surely we may pause before this dental professional pen and weigh its teachings, although "the waiter begs to offer an apology for this disconnected answer, for it could only be hastily dictated at different times when he had a little leisure." This "disconnected answer," as I read it, seems to be largely a eulogium of what is styled in the paper the "Black methods" of filling teeth, and a medium for bringing prominently into view an extensive private practice, eighty per cent. of which consists of the removal of old fillings (how old these fillings are is not stated), but some of them are acknowledged to be "very beautiful," but still "requiring to be replaced by fillings of a somewhat different character." Dr. Wedelstaedt says, "Since the arrival of the essayist's paper" (probably not over a month) "I have seen no less than a hundred patients for whom it will be necessary to replace a large number of fillings that have cavities of decay around them which were placed in the proximal surfaces of their teeth, and all these cavities of decay have been caused by opportunities which were left by the previous operator. In not one case was there a practical application of the extension for prevention theory."

Let us call to mind what it may mean when one man in ordinary practice, in the space of possibly four weeks, sees one hundred new patients,—an average of four to six per day,—"with extensive decay around the fillings of other operators."

Eighty per cent. of the efforts of dentistry, if we accept the statements of Dr. Wedelstaedt, are failures. I quote his words:

"Full eighty per cent. of the work I make is the removal of and the replacing of poorly made fillings which have failed on account of non-extension for prevention."

Hence eighty per cent. of the time given to dentistry is worse than wasted; and it thus becomes a cruel, unavailing infliction of suffering, a waste of time and means, and a useless exhaustion of nerve energy and of physical strength. In the face of all this claimed weakness of dentistry, our author and teacher is clamoring for another change in *mechanical methods* with which to cure defects having bacteriological and chemical causes. Unqualified condemnation is heaped upon what he calls "the methods of 1875;" and yet what those methods are, in view of the varied and independent styles of operating, it would probably be difficult for any one to explain. No distinction is drawn between the tooth-saving operations of such men as the late Marshal H. Webb, William H. Atkinson, McKellops, of St. Louis, Frank Abbott, of New York, and of many still in service, as Darby, Jack, Allen, Daboll, and many others, and the merest tyro.

And this quite prepares us for the climax,—the unique classification of dentists by our author. He says, "The members of the dental profession at the present time are divided into two classes: first, those who individualize their *work, build* [!] their cavities after some definite plan, systematize their *work* [!], and have *some idea regarding what they do;* and second, those who fill holes."

"It is a simple question, and let each answer for himself: To which class do *you* belong? I ask that you consider this question and also these two closing paragraphs."

We are more or less familiar with the "building" of the complicated towering structures of the present; with the "building" of locomotives, bridges, and other great works, but it seems new to think that any of the dental profession are engaged in "building cavities" or even "filling holes." That a dentist should "systematize his work and have some idea regarding what he does" does not seem a high standard of culture or one indicative of specially exalted attainments for men practising a profession; but this is the professional standard to which, according to this teacher, we are to attain.

The "closing paragraphs" are as follows: "It is now well understood that filling-material without cavity preparation is not a potent factor in saving teeth, but *filling-material in connection*

*with cavity preparation* is the most potent and only one for intelligent men to take into consideration in discussing any theory relating to the *care* and *salvation* of the human teeth. Scientific men recognize this as one of the fundamental principles and the advance and progress of dentistry depend wholly upon the practical application of this theory." I have put some of the matter quoted in italics the better to contrast its force and meaning.

According to our author we must desist from "discussing any theory relating to the care and salvation of the human teeth," which does not make "filling-material, in connection with cavity preparation, the most potent factor and the only one for *intelligent* men to consider."

Must the dental profession accept the dictum of such teaching or risk the ostracism of "scientific" or even "intelligent" men? In view of the limited horizon of such professional vision and the slavish subjection of dentistry to its teachings, is it any wonder that we are regaled with such editorials as that of Dr. Truman, which appeared in the INTERNATIONAL DENTAL JOURNAL for June, 1901? He says, "We are floating in shallow waters. . . . It would seem that we have not been doing all that is possible to meet changing conditions. . . . It is quite evident that all this means for dental colleges a more stringent method, if not a new standard of training."

It may be well here to recall the fact that Dr. H. C. Wood, a professor in the same University with Dr. Truman, has declared the D.D.S. to be "a badge of partial culture."

At the risk of being considered "unscientific" or "non-intelligent," I propose this evening to direct your attention to a subject which I consider of far greater importance to the profession and to our patients than the matter of "extension for prevention."

It may seem presumptuous to intimate that in this subject of Oral Prophylaxis, when fully comprehended, are matters of a magnitude to radically modify the present thought and conception of dentistry, and to greatly change the present methods of practice. To advance a step farther and suggest that there is hidden away under the *débris* of the oral cavity secrets of greater importance to the comfort and welfare of civilization than the great discovery of vaccination by Jenner in 1796 will doubtless seem as a magnification verging on absurdity. And yet we venture to predict that the future of dentistry will disclose this as a truism.

In the discussions of your society following the paper of Dr. F. L. Bogue one gentleman is reported as follows: "But to view this subject simply from the acid stand-point, and to say that environment only is to be considered, is not a fair statement of the question. The physical and chemical make-up of the tooth must in the nature of things equally influence the action that takes place, and as these vary in relative value in different cases, they are not constant factors;" and after further discussion involving several paragraphs the summing up is as follows: "And so this paper of Dr. Bogue goes a great way, I think, to support the views of so many of us, that there is something else besides environment to be considered in seeking a full solution of the philosophy of the decay of teeth." If "something else besides environment" is "to be considered in seeking a full solution of the philosophy of tooth-decay," surely nothing else is needed in seeking the philosophy of *prevention* of tooth-decay. It is change of environment. The summing up of the remarks of another is reported as follows: "When we arrive at conclusions based upon all the facts we are able to obtain, we will probably begin to consider these conditions from the view-point of specialists in medicine, and look for remedies which will influence oral fluids." This reminds me of a discussion along these lines at the Tri-Union Meeting in Richmond, Va., in May, 1900, at which one gentleman strenuously maintained that the proper thing for dentistry is to teach physicians; for they, "having charge of the little stomachs of the children, could so prescribe for them as to make good teeth."

Dentists teach physicians to prescribe for the making of good teeth! With oral fluids changing every hour of the day in health and disease in each individual; with mucus, acid, and saliva from three sets of glands, varying in character, now neutral, now alkaline, and now acid! Remedies to influence these conditions for the benefit of the teeth! What are the remedies? When administered? Under what circumstances? and How? Is this a proposition designed to be seriously considered as within the bounds of probabilities? Or is it some gossameric web of the imagination, floating far above earthly possibilities in an attenuated ether of a vague, dreamy nothingness?

There are three specially important subjects involved in the discussion of oral prophylaxis,—viz., Dental Caries, Alveolar Development, and Pyorrhœa Alveolaris,—all of which I shall be glad to consider with you if time and your patience permit. First, as to the discoveries and bearings of treatment in their relations to dental caries.

We all know what a perplexing theme the cause of tooth-decay has been in the profession in the past; how many theories have arisen and, shining for a time, have vanished "as a dream when one awaketh." There is the old chemico-vital theory, the electro-chemical theory of Dr. Palmer, in which there is a modicum of scientific truth, and the more advanced theory of Dr. Miller, to whom for its elucidation the profession is greatly indebted. The theory of Dr. Miller may be classed as chemico-bacteriological; decay being ascribed to the presence of bacteria and the chemical action of their products.

It is not my purpose to attempt to answer the question, What is tooth-decay? but we may properly consider one near of kin, but more practical, more closely identified with our interests, and altogether more important,—viz., How do teeth decay?

In previous writings, notably a paper read in 1898, the author has persistently maintained that tooth-decay always has its origin from the external surface of the tooth; if in the crown of the tooth, it begins in the enamel; if the parts are devoid of enamel, it begins at the surface of the dentine. Re-solving the enamel until contact with dentine is gained, it expands and proceeds along the lines of the tubules in the direction of the pulp. The progress of this process of chemical tooth-solution, which we call decay, is hindered and opposed by two conditions only,—namely, the constituency and consolidation of the dentine and the vital energy, a force often scarcely appreciable, interposed through the existence of the living pulp. It would seem that if any feature of tooth-decay is reasonably well defined, *this* is that feature; and yet I notice that this society has recently discussed this very proposition; and that not seemingly with any hesitancy or doubt, but with the full assurance of a settled finality.

To give emphasis to what is to be said on this subject, I will quote a paragraph from the discussions on the paper of Dr. Bogue, INTERNATIONAL DENTAL JOURNAL, May, 1901, page 335: "There is one remark in the discussion of the subject by Dr. Bogue to which I would like to take a slight exception,—namely, that teeth which are thoroughly clean will not decay. Clinically, I know of apparently clean teeth that have decayed, teeth which have been kept so absolutely clean that it could not be said that they were not so. I have attributed these cases to the fact that there is something in the enamel that invites decay that is not apparent on the surface. I only state it as an exception to the rule. I believe in general that clean teeth do not decay; in fact, I have very radical opinions on the subject." When a gentleman of the standing and attainments of Dr. Allen can talk of "apparently clean teeth that have decayed, because of something *in the enamel* that invites decay," we are forced to the conclusion that there is something radically wrong, *not* in tooth-enamel, but in the present thought and methods of dentistry.

Were we required to enunciate the fundamental principle governing treatment in a case of surgery, we would unhesitatingly say, first look for the cause, then endeavor for its elimination. This principle is as good and as applicable in dentistry as in surgery.

And in reviewing this matter we assume with confidence that the *beginnings* of decay are at the surface of the tooth, and that the agent or cause of it, whatever we may decide it to be, must reside in the menstruum in which the tooth is perpetually enveloped.

How, then, shall we interpose a sure, safe, and effectual preventive?

Prior to the publication of the paper, "Prophylaxis in Dentistry," in 1898, there were a few feeble, sporadic suggestions wholly in the direction of germicidal washes, but practical, beneficial results were entirely wanting. How could there be beneficial results from the occasional use of a doubtful germicide when the one and only remedy is the entire, positive, and frequent removal from tooth-surface of all matter inimical to tooth-life as well as to its composition?

Is not this in accord with reason and careful observation? And if so conceded, shall it be cast aside and rejected, as has been the teaching in some quarters, because it has not been ground in the mortar of a chemical laboratory or scrutinized under a high-power microscope?

Let us make a simple indisputable test. A devitalized tooth, sometimes, but improperly, called a "dead tooth," is generally conceded to be subject to more rapid decay in the mouth than a similar tooth with a living pulp. Let a pulpless, decayed tooth be removed from a mouth where the environments are such that re-solution is rapidly taking place, and let it be placed in water, alcohol, or glycerin, or simply exposed in the air, and we know that decay is immediately arrested and that further disintegration results only with the lapse of years.

It is wholly unnecessary to institute a process of artificial decay to prove the fact that when a tooth, bad or good, hard or soft, is removed from its environments,—from the menstruum of the mouth,—decay is at once arrested. The discovery and enunciation of the important fact that enforced and systematic change in the environment of the teeth in the mouth will prevent decay and carry with it many other beneficial results is new,—new in essence, new in conception, and new in its elaboration,—and results wholly from clinical investigation and experimentation.

It would probably be of interest to speak of the conditions and necessities which led up to these discoveries, but we may more profitably spend time in the study of what constitutes change of environment for the teeth, and what the process, as herein outlined, really does for them and for contiguous structures; the manner in which it is accomplished, and how it is maintained.

Treatment of the teeth for complete change of environment, briefly stated, consists in thorough removal, at frequent and regular intervals,—once a month has thus far proved most satisfactory, —of all accumulations, whether solids, inspissated excretions, semisolids, or bacterial formations, from all the exposed surfaces of the teeth, leaving the enamel, or whatever of the tooth may be exposed, thoroughly polished and thus in the best condition to void hurtful deposits and equally to favor all efforts of the patient in the direction of cleanliness.

It is readily demonstrable that to secure and maintain true cleanliness in the mouth, even on the part of the most painstaking, is impracticable if not impossible, without the direction and assistance of an expert and intelligent operator.

There are calcific deposits constantly increasing; the more immediately hurtful acidulated bacterial accumulations; inspissated mucus retaining decomposing particles of food and furnishing most favorable conditions for bacterial culture and the retention of excretions, not alone from the gum margin, but from the whole gum surface. Besides these, there are irregularities; formations and positions inaccessible to all ordinary methods of cleansing which implies the perpetual retention of matter inimical to the teeth and gums. These injurious accumulations with their equally injurious emanations, hitherto overlooked and disregarded by physician or dentist, are not only causes of decay, but are equally causes of absorption of alveolar structure and recession of gums. These latter conditions are far more to be dreaded than simple decay.

Recognition will yet be made of the important fact that to the presence of foreign matter on and about the teeth, rather than to the quantity of it, the beginnings of decay and pyorrhœa are wholly attributable; and the deleterious influence of a breath perpetually loaded with offensive emanations from this source, especially during seasons of salivary inactivity, as in sleep, will, we believe, ere long be disclosed as an important factor in many pulmonary and digestive disorders, and will be taken account of in medical diagnosis and treatment.

An interesting series of experiments to determine the precise conditions under which disease germs are carried by droplets of saliva when a person speaks or coughs or sneezes is described by Hermann Königer in the *Zeitschrift für Hygiene und Infectionskrankheiten.* The main results are given in an abstract in the *Revue Scientifique,* Paris, August 14, 1900:

"The author has been able to assure himself that in an apartment where there was no appreciable current of air, a person speaking, coughing, or sneezing could scatter germs to a distance of more than seven metres (twenty-two feet). The dissemination in speaking is different in different individuals, and the germs scattered abroad through the air by means of these droplets remain in suspension for only a short period. These droplets are really microscopic balloons having in the centre a bubble of air. The dissemination of these droplets, with germ-originating capabilities and tendencies, is most marked during coughing and sneezing. The dissemination by means of droplets is especially to be feared in case of small micro-organisms, such as the germ of influenza, of plague, and of pneumonia. The *bacillus prodigiosus* and the *bacillus mycoides* is not carried as far, and the danger of infection is consequently lessened.

"The more pathogenic microbes the mouth contains the greater the danger. Washing the mouth has the effect of decreasing the number of diphtheritic bacilli susceptible of being detached. Placing the hand or a handkerchief before the mouth prevents the emission of droplets charged with tubercle bacilli. In cases of pneumonia it would be necessary to wear a mask in front of the mouth. During a surgical operation no one present should speak. Measures might, of course, be multiplied indefinitely as suggested by this important idea of the scattering of microbes by droplets of saliva."

In view of the fact, as herein set forth, of the imminent and ever-present danger of infection from the inhalation of bacilli-producing droplets of saliva from mouths almost universally infected, is it not time for dentists and medical men to awake to the importance of enforced and positive cleanliness for mouth and teeth? The prophylactic treatment advocated in this paper contemplates the absolute and positive removal of the unseen, but real, bacterial plaque present in some situations on every untreated set of teeth, as well as the removal at frequent intervals of gum exudation, heretofore unsuspected and untouched, and the frequent and perfect polishing, by hand methods, of all exposed tooth-surfaces.

The establishment and maintenance of these conditions in the mouth has been found not only possible, but the feasibility of it has been fully demonstrated.

The process has been more commonly alluded to as cleaning teeth, but there is a wide distinction between the ordinary methods of "cleaning the teeth" and this system of prophylactic *treatment*. The difference is, first, in appliances and methods; second, in extent and thoroughness of the operation; third, in the persistence and frequency of the treatment; fourth, in the object sought and the results attained,—the prevention both of decay and pyorrhœa.

When necessary, scalers should be used for the removal of solid deposits and such mucous concretions as may have been the means of softening or causing a partial decalcification of cervical enamel; and this should be perfectly attained. Following this, the teeth should be *thoroughly polished on all exposed surfaces,*—the labial, buccal, palatal, lingual, mesial, distal, and, in cases of developing teeth, the occlusal as well. The hand-polishing with stick and pumice should reach to every exposed portion of the tooth, and be continued until the touch, which can be educated in this matter to distinguish better than the eye, gives evidence of thorough cleansing and polishing. The operation is best done with properly shaped orange-wood sticks charged with powdered pumice-stone. The prepared orange-wood is most conveniently handled and carried to positions desired by an efficient and properly shaped porte-polisher.

We believe there is no such thing as "a positively clean tooth," unless it has been made so by some special artificial process of cleansing.

The grit of not too finely powdered pumice-stone has been found best adapted for removing viscid mucoid accumulations and for polishing enamel surfaces; and what is even more important,—and now notice,—the friction of the stick and pumice as applied by hand—*for power-polishers should never be used*—seems to excite or stimulate the vital forces of the tooth to increased activity in the removal of waste and the deposit of new and better material. The effect has been likened to massage treatment for muscular tissue.

The benefits resulting from this treatment are most marked, and extend to all parts of the tooth.

Whilst the deciduous and young permanent teeth are most responsive, all classes of teeth, and teeth of all ages, are peculiarly and positively benefited, as is plainly shown in them after a few months of regular and careful treatment.

The benefits are not to the enamel or treated surfaces alone, but results are equally pronounced in pericementum, gums, the alveolar structure, and apparently in the dentine and cementum. There are striking exhibitions of change and improvement of color in the enamel, while it is apparently taking on from month to month a firmer texture and a more compact resisting structure. The change in color is from an opaque, old-ivory appearance to that of clear, translucent, polished enamel, the whole giving the appearance of increased density and general improvement, denoting decay-resisting structure; the apparent stimulation from the treatment rapidly changing the color of the tooth, diminishing its sensitiveness, both externally and internally, and greatly improving its quality; changes which have impressed and astonished the author as perhaps no other results from operations on the teeth or in the mouth have ever done.

But let it be clearly noted that our claim is that, whilst in this endeavor after change of environment the teeth are rendered immune, this result is by no means the only benefit, if, indeed, it is the most important.

Unlooked-for outgrowths from this treatment in other directions have been both surprising and satisfactory; developments have been so rapid as to make it difficult to keep pace with them. Again and again I have been compelled to advance what I had considered to be the extreme boundary lines into new territory.

Starting with the discovery of the true method for the prevention of tooth-decay, it quickly extended into the domain of tooth-betterment. There is an evident stimulation of the life forces of the tooth in this treatment, both through the pulp and through the pericementum and its adjuvants. The pulp is stimulated to the work of taking up the old stagnant, color-giving matter, and to the laying down into a more perfect organization of new, health-giving elements. The unsightly yellow in the crown and the dark brown so frequently found in the cervical dentine of exposed roots are replaced by the clear, translucent matter, best comprehended under the term tooth-color. I have seen in several instances, as a result of this treatment, the irregular unsightly white spots on labial faces of front teeth, a condition indicative of interrupted nutrition between dentine and enamel, resume a normal life-like appearance; and in a greater number of cases the dark brown, in root-exposed cervical dentine, replaced by matter of normal color.

### INFLUENCE ON GUM TISSUE.

A constant and by no means inconsequent result of the treatment is a decided change of condition in appearance and in fact, of the whole gum tissue. This change is more quickly noticeable than that in the teeth themselves. Relieved of the irritation due to the presence of the toxic matter at the cervix, the gums begin at once to lose the abnormal sensitiveness which often attaches to them, as well as the dark, congested appearance, indicative of the presence of an irritant, and to take on, especially over the alveolar process, a beautiful and permanent *striation*. Improvement in the festoons is equally noticeable, they assuming a more regular and pleasing appearance; there is change for the better in the whole aspect of the mouth.

### BENEFITS TO ALVEOLAR STRUCTURE.

Of the better, more regular and harmonious alveolar development resulting from this treatment I do not care now to speak with great emphasis, and only from the clinical stand-point. As the larger percentage of my patients who are under this treatment are in the period where the alveolar structure is endeavoring to make room for developing teeth (ranging from four to twenty years of age), the opportunity for observation on this point has been of a fairly satisfactory nature.

In every case where the eruptive process has been specially noted, from the sixth-year molar to the wisdom-tooth, there has been apparently an easier and a more natural eruption of the tooth, accompanied with less irritation to the surrounding parts; the expansion of the alveolar structure has been natural and proportionate to the room required for the erupting teeth.

In several instances where, from the crowded condition of the jaw, development, especially of the cuspid teeth, seemed to have been arrested, the relief afforded by this treatment—the frequent removal of obstructive matter on the teeth and from about the gums of the necks of the teeth by hand-polishing—has induced a resumption of the retarded eruptive process, and the teeth are now steadily and satisfactorily pressing their way into normal position.

I am more and more convinced that the practice of early interference with protruding cuspids, through other means than endeavors to induce alveolar expansion, is radically wrong and to be discouraged.

This treatment is greatly beneficial to the general health, removing as it does at regular intervals the excretions of gum and alveolar tissue, as well as the viscid mucoid accumulations gathered especially through the mischievous working of nocturnal mucus. It prevents the introduction of much solid toxic matter into the stomach and lungs; it greatly relieves the breath of offensive odors and of disease-giving bacterial culture, and maintains a condition of cleanliness for the mouth unattainable by other means.

To the undisturbed presence of toxic matter on and about the teeth we unhesitatingly charge the beginnings of all gingivitis and all pyorrhœa; hence it should be the true preventive, as it is when properly directed, the one remedy for this dread disorder.

